# A perfect storm: acute portal vein thrombosis in a patient with severe dengue and hemorrhagic manifestations—a case report

**DOI:** 10.1186/s43066-022-00233-9

**Published:** 2022-12-27

**Authors:** Carlos Eduardo Ruiz Gonzalez, Juan Daniel Criado Villamizar, Yuderleys Masías León, Diego Fernando García García, Katherine Tatiana Centeno Hurtado

**Affiliations:** Universidad Industrial de Santander, Bucaramanga, Colombia

**Keywords:** Portal vein thrombosis, Dengue, Thrombocytopenia

## Abstract

**Background:**

Dengue constitutes a public health problem in endemic regions. The clinical course can range from asymptomatic to severe expressions. Hemorrhagic manifestations are the most frequently reported complications; on the contrary, thrombotic complications are unusual.

**Clinical case:**

We present the case of an adult patient who presented hemodynamic instability, severe thrombocytopenia, and positive serology for dengue, in whom acute portal vein thrombosis was documented. The possible pathophysiology of thrombocytopenia and thrombosis in dengue is discussed, as well as the dilemmas regarding the treatment of associated hemorrhagic and thrombotic manifestations.

**Conclusions:**

The present case brings up the importance of considering the possibility of thrombotic events in patients with severe dengue. A high degree of suspicion, close assessment of hemostatic function, and quality supportive care are essential to improve outcomes. To our knowledge, this is the first report of dengue-associated portal vein thrombosis.

## Introduction

Dengue virus is transmitted mainly by mosquitoes of the *Aedes aegypti* species, recognizing 4 different serotypes (DENV 1-4). It especially affects tropical and subtropical regions, where it has increased more than other communicable diseases, with an increase in the incidence of 400% between 2000 and 2013 [1].

In Colombia, dengue infection is a public health problem; during 2019, 119,840 cases and a total of 79 deaths were reported [2]. The clinical manifestations of the disease can range from mild symptoms to more severe but infrequent manifestations such as septic shock and coagulation disorders [3], where hemorrhagic events predominate. Cases of thrombosis are exceptional and only a few have been reported in the literature [4–6].

We present the case of an adult with dengue who presented with signs of dengue shock syndrome complicated by extensive acute thrombosis of the portal vein without other associated prothrombotic factors. To our knowledge, it is the first described case of portal vein thrombosis associated with dengue.

### Case report

In October 2019, a 51-year-old woman from a rural area of Santander, Colombia, was admitted to our unit with a story of fever and shivers for the past 5 days associated with epigastric pain, emesis, diarrhea, and an episode of hematemesis. Previous medical history included hypertension and dyslipidemia, and she was taking enalapril, hydrochlorothiazide, atorvastatin, and ASA.

Examination revealed hemodynamic instability, tachycardia, hypotension requiring vasopressors, delayed capillary refill time, and dehydration. Petechiae were identified on the skin of the extremities. Cardiopulmonary auscultation documented bilateral basal rales and palpation of the abdomen, painful hepatomegaly (4 cm).

**Fig. 1:**
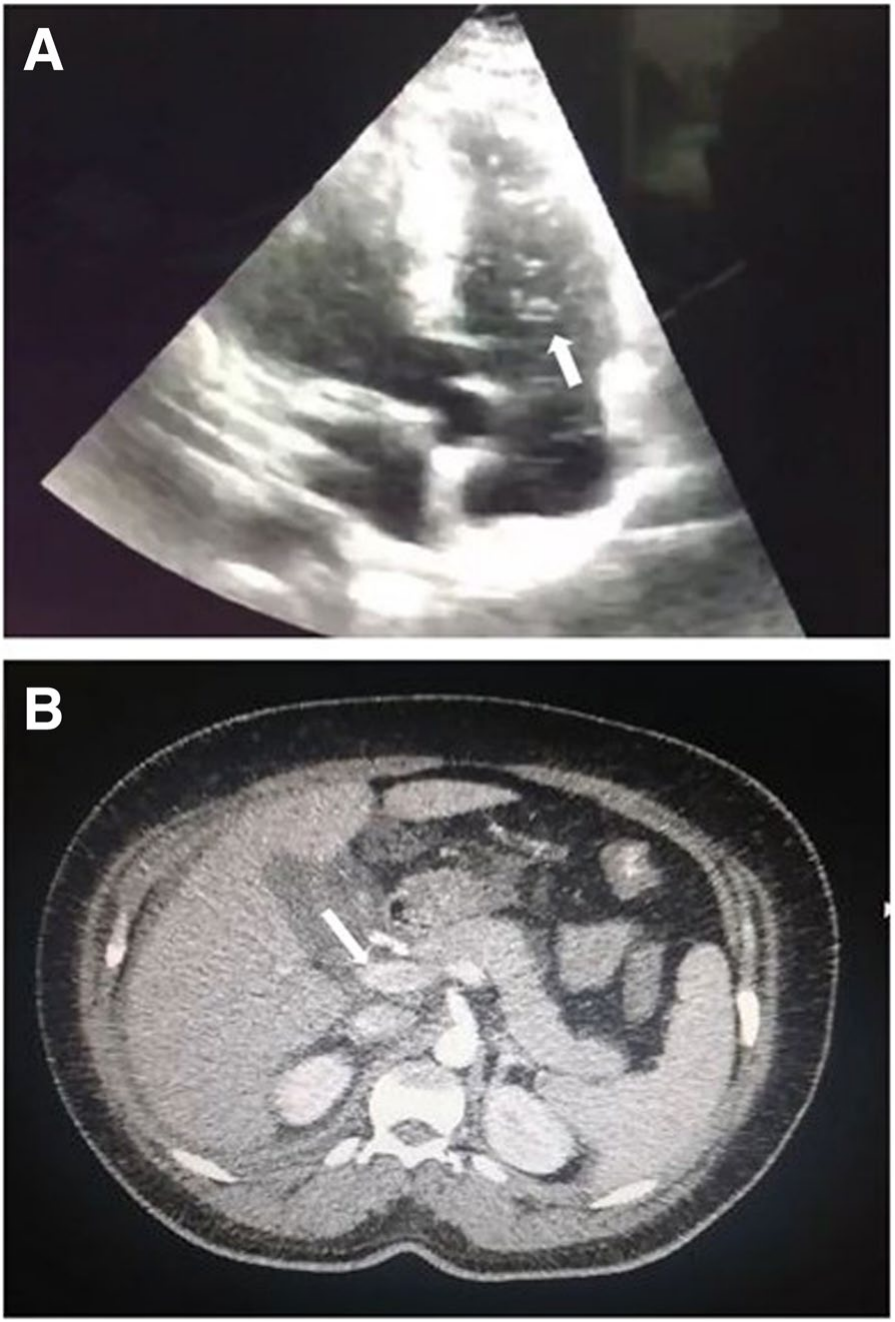
Diagnostic Images performed. **A** Transthoracic echocardiogram showing the presence of bubbles in the right chambers (white arrow). **B** Contrast-enhanced abdominal tomography showing acute non-cirrhotic portal vein thrombosis: partial enhancement of the portal vein, showing an image compatible with a thrombus inside (white arrow)

Her blood count showed pancytopenia with severe thrombocytopenia (7000 cc/mm^3^). Liver function tests revealed transaminase elevation (50 times above its normal value), LDH> 1000, normal bilirubins, and normal alkaline phosphatase suggesting hepatocellular injury with an ischemic component; the PTT was prolonged without PT alteration (See Table 1). The chest X-ray and abdominal ultrasound showed evidence of leakage with free fluid in the abdomen and pleural effusion, and hepatomegaly was also noted. The presence of bubbles in the right cavities without evidence of shunt was reported by echocardiography, as a consequence, a Doppler ultrasonography of portal and mesenteric veins were performed as well as CT with portal venous phase, showing an image compatible with acute complete non-cirrhotic thrombosis in the proximal portion of the portal vein (see Fig. 1). She was serologically confirmed positive for anti-DENV-1 IgM by ELISA [7].

**Table 1:**
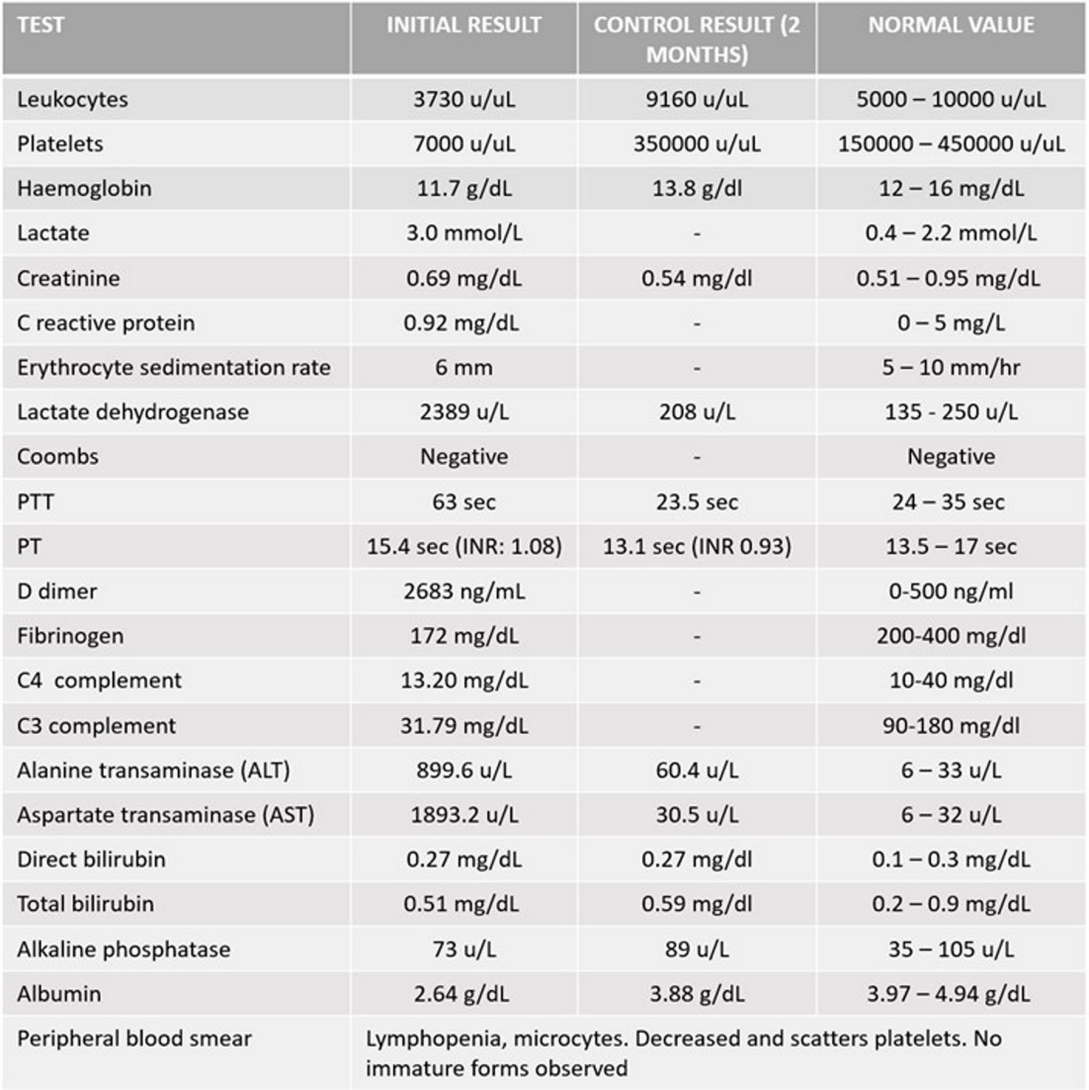
Laboratory tests performed on admission and at 2-month follow-up (control)

*PTT* partial thromboplastin time, *PT* prothrombin time, *INR* international normalized ratio

The patient required close monitoring in the intensive care unit with vasopressors for 11 days due to distributive shock. A presumptive diagnosis of severe dengue with secondary coagulopathy and thrombotic complications was made.

She received methylprednisolone and prednisone 1 mg/kg as maintenance for suspected ITP, and anticoagulation with LMWH was started when platelets reached >70,000 cells/mm^3^. She was treated with piperacillin tazobactam (4.5 g IV q.i.d.) for 7 days starting at the 6th day of thedisease.

The patient was further evaluated for additional causes of acute hemorrhagic fever syndrome with thrombotic events associated as well as prothrombotic systemic states (See Table 2). Cultures of the blood, urine, and stool yielded normal results. Malaria and Chagas tests in blood smear were negative. Hepatitis A, B, and C virus infections were ruled out serologically as well as CMV, HIV, and Leptospira infections. Screening for TMAs and inherited thrombophilia did not reveal any abnormality. She was screened for antiphospholipid syndrome, protein C and S deficiency, factor V Leiden, hyperhomocysteinemia, and ATIII deficiency. There was no evidence of hemolytic anemia, blood smear did not show abnormal cells, and ADAMTS 13 levels were normal. Complement fraction C3 was consumed and C4 was normal. ANAs, anti-dsDNA, and ANCAs were also negative. She was not tested for COVID-19 given that the first case reported in our country occurred in March 2020.

**Table 2:**
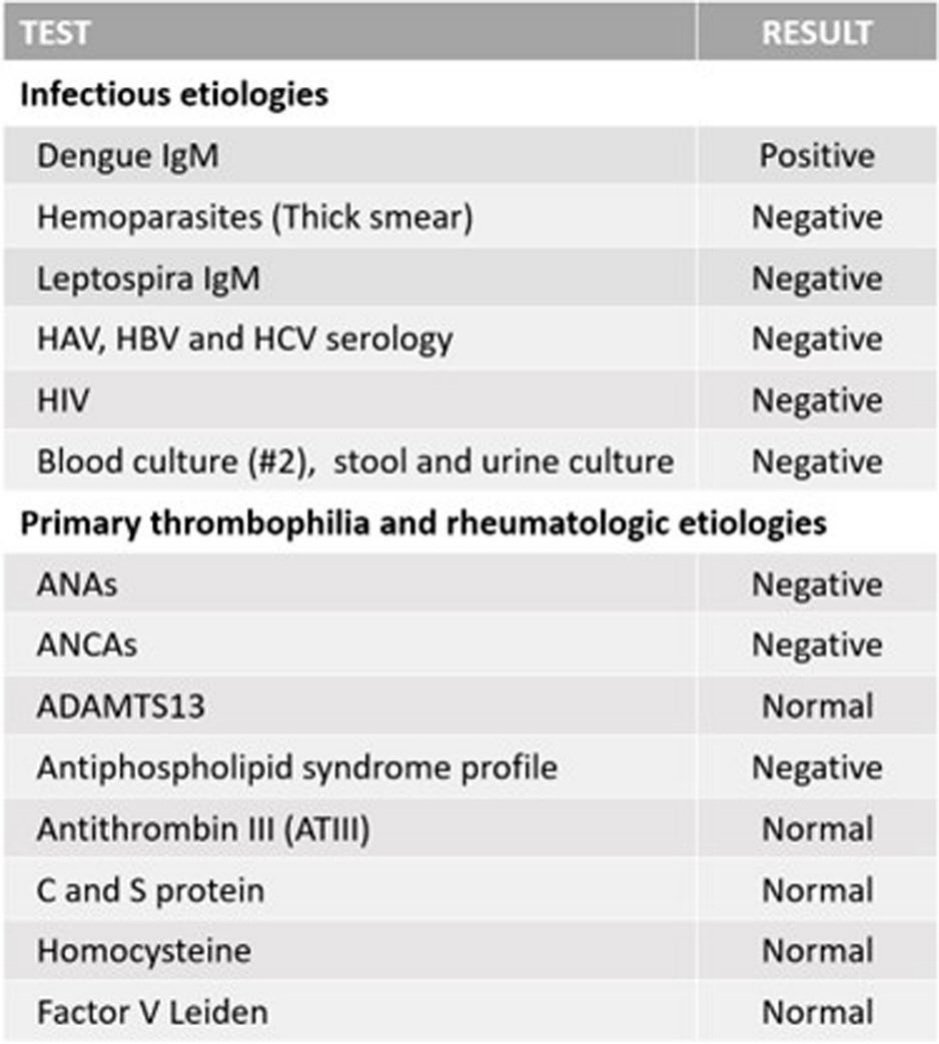
Prothrombotic state and acute non-cirrhotic portal vein thrombosis etiologies ruled out

*HAV* hepatitis A virus serology, *HBV* hepatitis B virus serology, *HCV* hepatitis C virus serology, *HIV* human immunodeficiency virus, *ANAs* antinuclear antibodies, *ANCAs* antineutrophil cytoplasmic antibodies, *ADAMTS13* A-disintegrin and metalloproteinase with thrombospondin type 1 motif, member 13

She underwent a control 8 weeks later, and Doppler ultrasonography of portal and mesenteric veins as well as laboratory initial alterations were completely normal. Steroid and anticoagulation therapy was discontinued after 3 months.

## Discussion

In the absence of liver cirrhosis or hepatocellular carcinoma, portal vein thrombosis (PVT) is a consequence of hypercoagulable states (local or systemic), myeloproliferative disorders, some inherited thrombophilia, abdominal surgery, and intra-abdominal infections are the best-defined etiologies. In the presence of thrombocytopenia and acute thrombotic complications, the diagnosis spectrum comprises a handful of entities of which HIT, APS, TMAs, and DIC are the most relevant ones.

Dengue coagulopathy is a rare cause of deep vein thrombosis and is scarcely reported. A number of subjacent mechanisms that could contribute to procoagulant states (in addition to hemorrhagic manifestations) have been studied in the literature. Endothelium damage is complex and comprises several pathways, and NS1 enhances indirectly the disruption of the endothelial barrier through immune-mediated stimulation of TLR4 resulting in the production of pro-inflammatory cytokines, complement activation, vascular leakage, and subsequent activation of endothelial cells with dysregulation of surface molecules (upregulation of adhesion molecules such as VCAM-I e ICAM-I and degradation of endothelial glycocalyx mediated by heparanase-1) [8–10]. NS1 also disrupts platelet function, increasing platelet adherence to endothelial cells and inducing platelet activation and aggregation via TLR4/MyD88 even in the presence of a subthreshold dose of ADP. This NS1-induced platelet activation/apoptosis could not only be related to thrombocytopenia and microthrombi formation but also to enhancing inflammatory response in dengue patients [11]*.*

Other procoagulant alterations include a decrease in ATIII, proteins C and S, and an increase in tissue factor and PAI-1 [12], mild prolongation of PTT, and moderate to a severe reduction in fibrinogen levels with loss of factors secondary to increased vascular permeability are also a frequent finding [3]; hence, it has been established that instead of causing a true disseminated intravascular coagulation, dengue infection can mainly activate fibrinolysis, causing a secondary activation of the procoagulant homeostatic mechanisms. Severe plasma leaking leading to dehydration and hemoconcentration, a characteristic of severe dengue, is also a well-known risk factor associated with thrombotic events.

It remains a mystery why thrombotic events are not reported more frequently even though the disease has low levels of natural anticoagulant proteins and higher levels of the main procoagulant and antifibrinolytic agents. It seems that, unlike what occurs in bacterial sepsis, the preserved or increased levels of thrombomodulin counteract the effect of the deficiency of anticoagulant proteins [13].

Da Costa et al. reported an incidence of 5.4% thrombotic events in patients with severe dengue with a total of 5 venous thrombotic events, 4 of the cases were DVT and 1 mesenteric venous thrombosis; nonetheless, it was not an isolated event since concomitantly *Escherichia coli* bacteremia, a well-known risk factor for portal vein thrombosis, was diagnosed [4]. Other thrombotic events associated with dengue described in the literature are mainly cerebral venous thrombosis and DVT; in many of these cases, an associated procoagulant factor was not found either [5, 6].

At the time of evaluation, our patient had signs compatible with dengue shock syndrome as well as laboratory abnormalities that suggested not only severe coagulopathy but also activation of the complement system, which has been posed on previous occasions as a key underlying event in DHF/DSS immunopathogenesis [14]. Even when it has been previously stated that DENV mainly activates fibrinolysis in the absence of a thrombotic stimulus, we propose that in our case the combination of dengue-associated coagulopathy and shock-derived dysoxia could have led to a true DIC. Although the patient had arterial hypertension and dyslipidemia, which are part of metabolic syndrome, a well-known prothrombotic-related syndrome, additional risk factors for the development of VT were excluded through judicious evaluation.

In addition to the above, the apparent response of thrombocytopenia to steroids also attracted attention, a phenomena infrequently described in some series, and which would support the presence of an immune mechanism [15].

Despite the multiple reports that have been made on dengue immunopathogenesis and the complex dysregulation of the immune system that takes place, there are no definitive recommendations that allow determining the scenarios in which patients can benefit from steroids or other immunomodulators [15]. Regarding treatment with anticoagulants, the data are anecdotal and extrapolated from experience in other pathologies with concomitant hemorrhagic and thrombotic risk.

Finally, it is worth noting that 4 of the 5 patients with thrombosis in Da Costa et al.’s study were older than 50 years as well as our patient, which leaves the question about the risk of age-related thrombotic events in patients with dengue infection [4].

## Conclusion

The present case brings up the importance of considering the possibility of thrombotic events in patients with severe dengue, especially if it is accompanied by dengue shock syndrome; these patients also have a higher risk of bleeding complications and bacterial infections (which in turn increases the probability of sepsis and DIC). A high degree of suspicion, close assessment of hemostatic function, and quality supportive care are essential to improve outcomes.

## Data Availability

Because it is a case report, data sharing is not applicable to this article as no datasets were generated or analyzed during the current study.

## Abbreviations

DENV: Dengue virus
ASA: Acetylsalicylic acid
LDH: Lactate dehydrogenase
PTT: Partial thromboplastin time
PT: Prothrombin time
IgM: Immunoglobulin M
ITP: Idiopathic thrombocytopenic purpura
CT: Computed tomography
LMWH: Low molecular weight heparin
DIC: Disseminated intravascular coagulation
TMAs: Thrombotic microangiopathies
ATIII: Antithrombin III
PAI-1: Plasminogen activator inhibitor-1
HIT: Heparin-induced thrombocytopenia
ADP: Adenosine diphosphate
APS: Antiphospholipid syndrome
DVT: Deep venous thrombosis
DHF: Dengue hemorrhagic fever
DSS: Dengue shock syndrome
